# Cost-effective and green additives of pozzolanic material derived from the waste of alum sludge for successful replacement of portland cement

**DOI:** 10.1038/s41598-022-25246-7

**Published:** 2022-12-05

**Authors:** O. A. Mohamed, A. A. Farghali, Ashraf K. Eessaa, A. M. El-Shamy

**Affiliations:** 1 Environmental Science and Industrial Development Department, Faculty of Postgraduate Studies for Advanced Sciences, Beni-Suef University, Beni Suef, 62511 Egypt; 2 Materials Science and Nanotechnology Department, Faculty of Postgraduate Studies for Advanced Sciences (PSAS), Beni-Suef University, Beni Suef, 62511 Egypt; 3 Nanotechnology Central Lab, Electronics Research Institute (ERI), Cairo, Egypt; 4 Electrochemistry and Corrosion Lab., Physical Chemistry Department, National Research Centre, El-Bohouth St. 33, Dokki, P.O. 12622, Giza, Egypt

**Keywords:** Materials science, Mathematics and computing

## Abstract

The major objective of this study was to examine the viability of using 5, 10, or 15 mass% of Activated Alum Sludge waste (AAS) instead of Ordinary Portland Cement (OPC) as a pozzolanic ingredient in concrete. This fundamental inquiry framed the investigation and OPC-AAS-hardened composites were studied to see whether they may benefit from inexpensive nanocomposites in terms of improved physical properties, mechanical strength, and resistance to heat and flame. The investigation set out to see how inexpensive nanocomposite might be put to use and the nanoparticles of CuFe_2_O_4_ spinel with an average size of less than 50 nm were successfully manufactured. Many different OPC-AAS-hardened composites benefit from the addition of CuFe_2_O_4_ spinel, which increases the composites' resistance to fire and enhances their physicomechanical properties at roughly average curing ages. Synthesized CuFe_2_O_4_ spinel was shown to have desirable characteristics by TGA/DTG and XRD. By using these methods, we were able to identify a broad variety of hydration yields, including C–S–Hs, C–A–S–Hs, C–F–S–Hs, and Cu–S–Hs, that enhance the physicomechanical properties and thermal resistivity of OPC-AAS-hardened composites as a whole. The composite material comprising 90% OPC, 10% AAS waste, and 2% CuFe_2_O_4_ has several positive economic and environmental outcomes.

## Introduction

As a result of the fact that it is regarded as a hazardous waste, alum sludge (AS), which is a by-product of water treatment plants, is unable to be disposed of in a landfill in a typical manner. Instead, the material must be transported to a specialized facility that is built for the express purpose of dealing with hazardous waste^1–3^. It is essential to dispose of solid wastes, particularly alum sludge (AS), as quickly as is practically practicable to reduce the number of potential threats that may be posed to the environment^4^. However, processed alum sludge, which is sometimes referred to as TAS, contains significant quantities of silica dioxide and aluminum oxide, which are the primary components of cement^5^.

As a result of this, Treated Alum Sludge TAS is a desirable alternative to the more traditional cement. Because of their ability to react with calcium hydroxide that is formed during the hydration of Ordinary Portland Cement (OPC), researchers in the field of concrete are becoming more interested in the practicability of recycling (AS) as a pozzolanic material with Portland cement^6^. This is because AS can interact with calcium hydroxide, which is the cause of this phenomenon. Because of the high pozzolanic activity it possesses, calcined water treatment sludge (also known as WTS) has recently been the focus of a few research studies^7^. These studies have investigated the possibility of incorporating calcined WTS as an additional cementitious material in cement-based products and evaluated how doing so would impact the mechanical properties of the cement^8–10^.

The results of the research indicated that the incorporation of drinking water sludge ash (DWSA) into Portland cement resulted in the development of hydrates that included aluminum. Ettringite and calcium aluminate hydrates (C–A–H) are two examples of hydrates that fall into this category since both hydrates may be found in Portland cement. This assertion is supported by the existence of a robust connection between the two variables in question. In recent years, nanotechnology has seen widespread application across virtually all areas of human endeavor to produce fresh uses. The industry of building and construction is particularly good at illustrating this trend's prevalence^11^. A lot of attention is being paid to the process of incorporating nanoparticles into cement materials. The goal of this process is to inspire new types of construction materials that have high efficiency and endurance under harsh environmental conditions^12–17^. These materials can be used in a variety of applications^18^.

This is something that can be done to produce new varieties of building materials that have these qualities. These components are potentially useful in a broad variety of settings due to their adaptability and versatility. Nano silica, nano iron, nano alumina, nanofibers, nano titania, and carbon nanotubes are some of the nanoparticles that are commonly used. Due to the characteristics that it possesses, ferrite is used in a broad variety of different applications as a magnetic semiconductor. In this context, “applications” refers to things like rotary transformers, noise filters, and multilayer ferrite chip components, among other things^19–21^. The electronic industry makes substantial use of ferrites, which are among the most important components of the sector's overall inventory. Recent research has been done to explore the effect that Fe_3_O_4_ spinel nanoparticles have on the durability of cementing materials. These investigations were carried out to find out more information^22^.

According to the findings, the incorporation of spinel nano-Fe_3_O_4_ improves not only the thermal resistivity of the materials but also their resistivity against the hostile anions that are present in the matrix. This is the case even though the incorporation of spinel nano-Fe_3_O_4_ does not improve the thermal resistivity of the materials^23^. As was just mentioned, many studies have been carried out and published to investigate the effect that nanoparticles have on the various properties that cementitious matrices present. These studies report their findings to better understand the relationship between nanoparticles and cementitious matrices^24–26^.

On the other side, there is either no research looking into the effect that CuFe_2_O_4_ spinel nanoparticles have or merely a very tiny number of studies looking into the effect that these nanoparticles have^27^. Because of this, the purpose of this research was to utilize low-cost synthesized CuFe_2_O_4_ spinel nanoparticles (CFs NPs) as an additive for boosting the mechanical properties and fire resistivity of hardened pastes made from Ordinary Portland Cement replaced by various ratios of activated alum sludge and to investigate the feasibility of the reuse of activated alum sludge for partially replacing Portland cement in construction materials, which helps in minimizing the environmental impact^28^.

### Corrosion of steel in cement

When steel is embedded in concrete, the carbonation or chlorides in the concrete are often the agents that cause the steel to corrode over time^29^. Carbonation is the process by which carbon dioxide from the air mixes with calcium from the concrete. This process is referred to by the word “carbonation”^30^. This is evidence that the pH of the concrete is decreasing, which in turn leads the steel to begin corroding^31^. Chlorides can be transferred from concrete to steel in a process known as chloride-induced corrosion^32^. This process can result in a significant acceleration of the pace at which corrosion occurs^33^.

According to the findings of the study that has been carried out up to this point, it has been postulated that there is a certain chloride threshold level that must be achieved before the start of a high corrosion rate^34^. Researchers have spent decades attempting to determine this chloride threshold level; nevertheless, the findings from their investigations have been highly variable^35^. This is likely the case given the large diversity of experimental conditions that were used, in addition to the fact that steel and concrete of varied quality were used in the experiments^36^. The amount of moisture that is already present in the concrete is an important factor that needs to be taken into consideration^37^. It is generally accepted, thanks to the high resistivity of dry concrete, that the rate of corrosion of steel in dry concrete is low^38^. This is because of the high resistivity of dry concrete. Extremely wet concrete has a corrosion rate that is comparable to that of moderately wet concrete due to the slow rate at which oxygen travels to the surface of the steel^39^.

When the moisture condition is intermediate, the rate of corrosion is high because the material has a relatively low resistivity and oxygen moves through it at a quick pace and this causes the rate of corrosion to be high. One strategy that might be used to cut down on the quantity of steel that corrodes in concrete is to make use of concrete that needs a significant amount of time to reach the chloride threshold level. This strategy has the potential to be applied^40^. One method that might be used to attain this goal is the application of a considerable layer of concrete. Other methods include employing thick forms of concretes that have a low water/cement (w/c) ratio and/or adding additives such as micro silica that may diminish porosity, which in turn slows the chloride transit rate. Both methods are examples of alternative ways since these approaches are instances of different approaches that might be used^41^.

There are currently several distinct types of cement that can be purchased from retailers. The European standard EN 197-1, which was produced by the European council for standardization in the year 2000, is used to discern between different types of cement based on how their chemical composition varies^42^. The European standard EN 206-1 (Standardization 2000), it is suggested that for concrete that is going to be exposed to the splash/spray zone of seawater, the concrete should have a water-to-cement ratio of 0.45 and a cement content of 340 kg cement by m^3^ concrete. This is because the ratio of water to cement determines the strength of the concrete, and the cement content determines how much cement is present in the concrete^43^. Because of this, the w/c ratio is used to determine the proportion of water to cement that is present in the concrete.

## Materials and methods

### Materials

The Blaine surface area of Ordinary Portland cement (OPC-type I) is determined to be 3495 cm^2^/g based on the calculations. The cement that was used by the participants of the study was supplied to them by the El-Sewedy cement company, which is in Al-Ain Al-Sokhna, Suez, Egypt. The processes that were used to purify drinking water ultimately led to the generation of a waste product referred to as water treatment plant sludge (WTPS). The water treatment plant samples (WTPS) that were acquired from the Beni-Suef facility and used in this investigation were obtained there. The WTPS was initially allowed to air-dry for 24 h in an oven set to 105 °C before being crushed. The alum sludge used in this study went through a procedure known as thermal activation, which was a part of the research process. To do this, dried WTPS was first heated in an electric furnace at a temperature of 500 °C for 2 h before being allowed to gradually drop down to room temperature^44^.

This investigation made use of a superplasticizer (SP) that was a modified polycarboxylate-based superplasticizer (PCsp) called Sika Viscocrete 5230 L. It has a specific gravity of 1.08 g/ml and was supplied by Sika Company in El-Obour City, Egypt. The evaluation investigated the effects of using an SP. SP superplasticizer is used not only as an agent for dispersing the nanoparticles but also to achieve the necessary level of workability in a variety of pastes. This is accomplished by utilizing the superplasticizer's dual-functioning ability to both disperse and work with the nanoparticles. The physical properties of the SP superplasticizer are broken out in further since its color is yellow–brown liquid, the percentage of solid residue is about 39.9%, the pH is ranged from 7.51 to 7.53 and finally, its specific gravity is about 1.08 g/ml.

**Figure 1:**
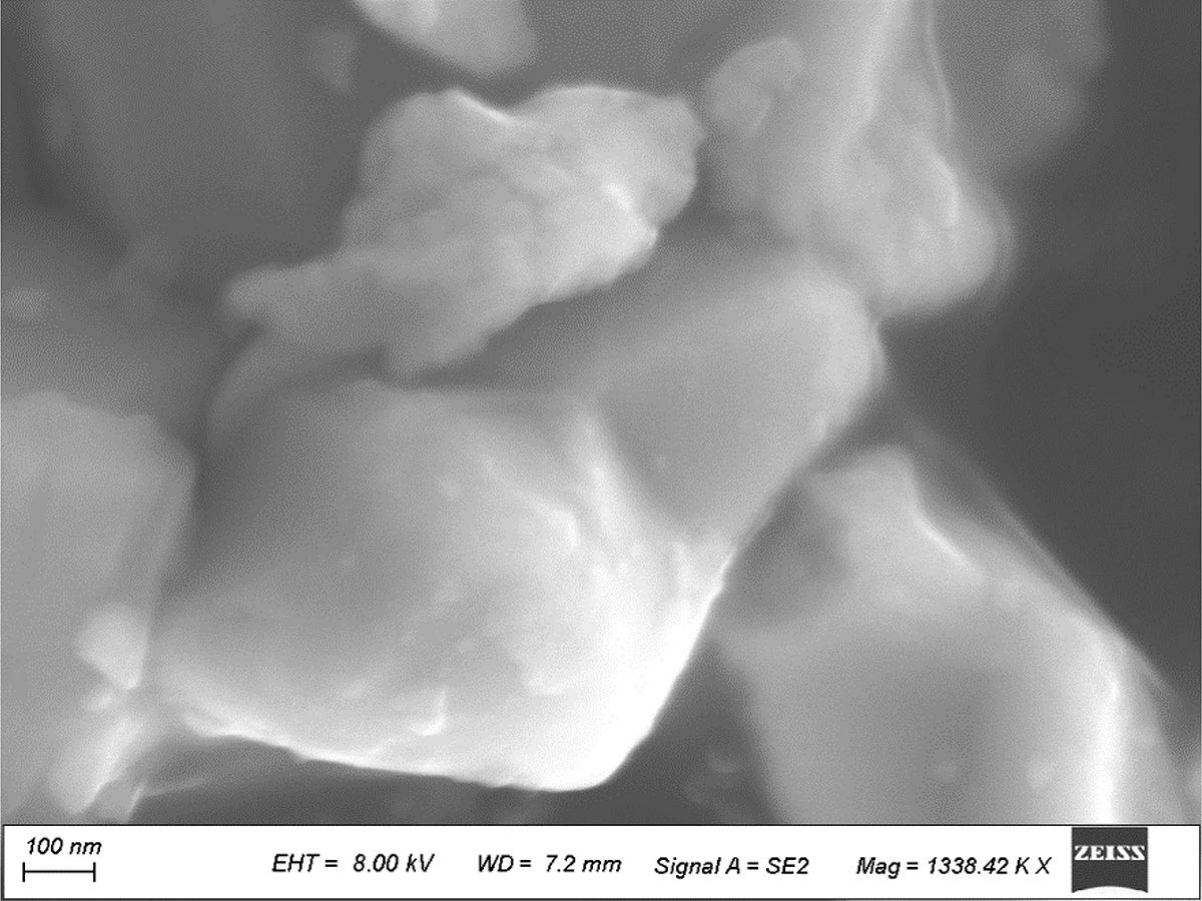
SEM Image showing the nanoparticles of CuFe_2_O_4_.

To determine whether the mixture was homogeneous, two moles of pure fine powder ferric acetate basic ((CH_3_COO)_2_·FeOH) were thoroughly combined in a ball mill for 6 h with one mole of copper (II) acetate monohydrate ((CH_3_COO)_2_·CuH_2_O)^45^. Following that, the mixture was dried at a temperature of 150 °C, and the sample was heated on a hot plate so that the acetates could be broken down more easily. To get copper ferrite nanocrystals, the sample was finally subjected to a 2-h-long heating process at a temperature of 100 °C. Using SEM, the CFs NPs that had been produced at a size of 200 nm were subjected to a mechanochemical degradation process, which resulted in the generation of CFs NPs that had an average particle size of less than 50 nm as seen in Fig. 1.

### Methods

There were several different types of OPC–AAS dry mixes that were created and includes both the components that made them up and the names that were assigned to those components. To guarantee that every dry combination was consistent, it was exposed to 8 h of mechanical mixing in a porcelain ball mill. This was done to ensure that the mixtures were perfectly even. This was done to attain complete uniformity in the environment. The incorporation of a range of different amounts in each of the varied mixtures resulted in an improvement of the pastes (CFs NPs). Following the addition of the CFs NPs, the aqueous solution that contained the superplasticizer (which contained 0.30% of Super Plasticizer (SP) by mass of solid) was combined with the total quantity of mixing water, and the resulting suspensions were sonicated at a temperature of 25 °C for 1 h. After mixing the dry ingredients with 0.27 parts water to 1 part cement, the resultant paste was used in several different tests^46^.

When the dry materials were mixed with the necessary amount of water (which, in the case of the creation of nanocomposites, contained the dispersed CFs NPs) and the mixing technique was continued for approximately 4 min, a variety of pastes were produced. Examples of each paste were fabricated with galvanized steel that had been molded into the shape of a cube, and the dimensions of these cubes were 2.25 cm on each side. The freshly molded pastes were kept for 1 day in an environment with a relative humidity that was lower than 100% to achieve the necessary level of final setting. Following that, the specimens in the form of cubes were placed in room-temperature tap water for 3, 7, and 28 days, respectively. During the compressive strength test, an apparatus that was made in West Germany by Ton-industries was used; at each stage, the average value of all three cubes was recorded. According to the method that had been described in the past, the dehydration process of the broken specimens was stopped by using a stopping solvent that was created by combining methanol and acetone in a volume ratio that was equal to 1:1.

After being made, the samples were held at temperatures of 80 °C for 3 h before being moved to a desiccator (which included soda-lime and CaCl_2_) and stored there until the time came for evaluating them. Total porosity (TP), bulk density (BD), and water absorption (WA) values were taken at regular intervals 46 to track the development of various essential physical characteristics. The temperatures at which the samples were stored varied from 75 to 80 °C. The values of total porosity, bulk density, and water absorption were tracked using these measurements after they were collected. According to the standards that have been set by the ASTM (C140 and C150). When a BD, TP, and WA are needed to compute, Eqs. (1), (2), and (3), respectively, should be used.

1 $${\text{BD }} = \frac{{{\text{W}}c}}{{{\text{ Wa}} - {\text{W}}b}}$$

2 $${\text{TP }} = \frac{{{\text{Wa}} - {\text{W}}c}}{{{\text{Wa}} - {\text{W}}b}}*{ 1}00$$

3 $${\text{WA }} = \frac{{{\text{Wa}} - {\text{W}}c}}{{{\text{W}}c}} *{ 1}00$$

where W_a_, W_b_, and W_c_ are the weights of saturated, suspended, and dried (at 105 °C overnight) samples, respectively.

The samples that were used for the thermal resistivity test were submerged in room temperature tap water for 28 days, dried at temperatures of 80 °C for 1 day, and then put through firings at temperatures of three hundred, six hundred, and eight hundred degrees Celsius for 3 h at each temperature. The procedure for heating was carried out inside an electric furnace so that the material could be subjected to the needed higher temperatures. There were two ways that the burnt samples were cooled: the first set of specimens were allowed to cool gradually, while the second group was cooled quickly by being submerged in tap water. Both approaches were employed. Multiple techniques were used to ascertain each set of cooled specimens' respective compressive strengths. To evaluate the textural characteristics and phases that were created throughout the hydration process, selected specimens were analyzed using X-ray diffraction (XRD), thermal gravimetric analysis (TGA/DTG), and scanning electron microscopy (SEM)^47^.

#### Corrosion test

##### Weight loss measurements

The rate of corrosion was determined based on the assumption that it was uniform throughout the whole surface of the coupons. Using the following formula, we were able to determine the corrosion rate in mils per year (mpy) based on the weight loss^48^:

4 $$CR = \frac{W}{{\left( {D \times A \times t} \right)}} \times k$$

where W = weight loss in grams, k = constant (22,300), D = metal density in g/cm^3^, A = coupon area (inch^2^) and t = time (days).

## Results and discussion

### Material characterization

The XRD patterns previously carried out for activated alum sludge waste changed significantly after being subjected to calcination at a temperature of 500 °C (AAS). The findings of the XRD examination performed on the AAS's various components and minerals. The diffractogram reveals the presence of a significant quantity of the mineral quartz (SiO_2_), which is one of the primary minerals in AAS. Additionally, the diffractogram reveals the presence of the minerals albite and calcite to a lesser extent, as well as the mineral kaolinite, which demonstrates a pozzolanic property upon calcination. The presence of quartz during the mineralogical investigation carried out by AAS is thus evidence that the substance in question has an amorphous silica structure. Over a wide range of d-spacing (10–85°, 2 θ), revealing the amorphous structure of aluminum in the AAS, there is no significant crystalline-aluminum-related XRD peak seen. After 7 and 28 days of hydration, the PC, PC–2CFs, PC–10 AAS, and PC–10 AAS–2 CFs (Mixes A, ACFs3, and CCFs3, respectively) were subjected to thermogravimetric (TGA) and differential thermogravimetric (DTG) analyses, respectively.

**Figure 2:**
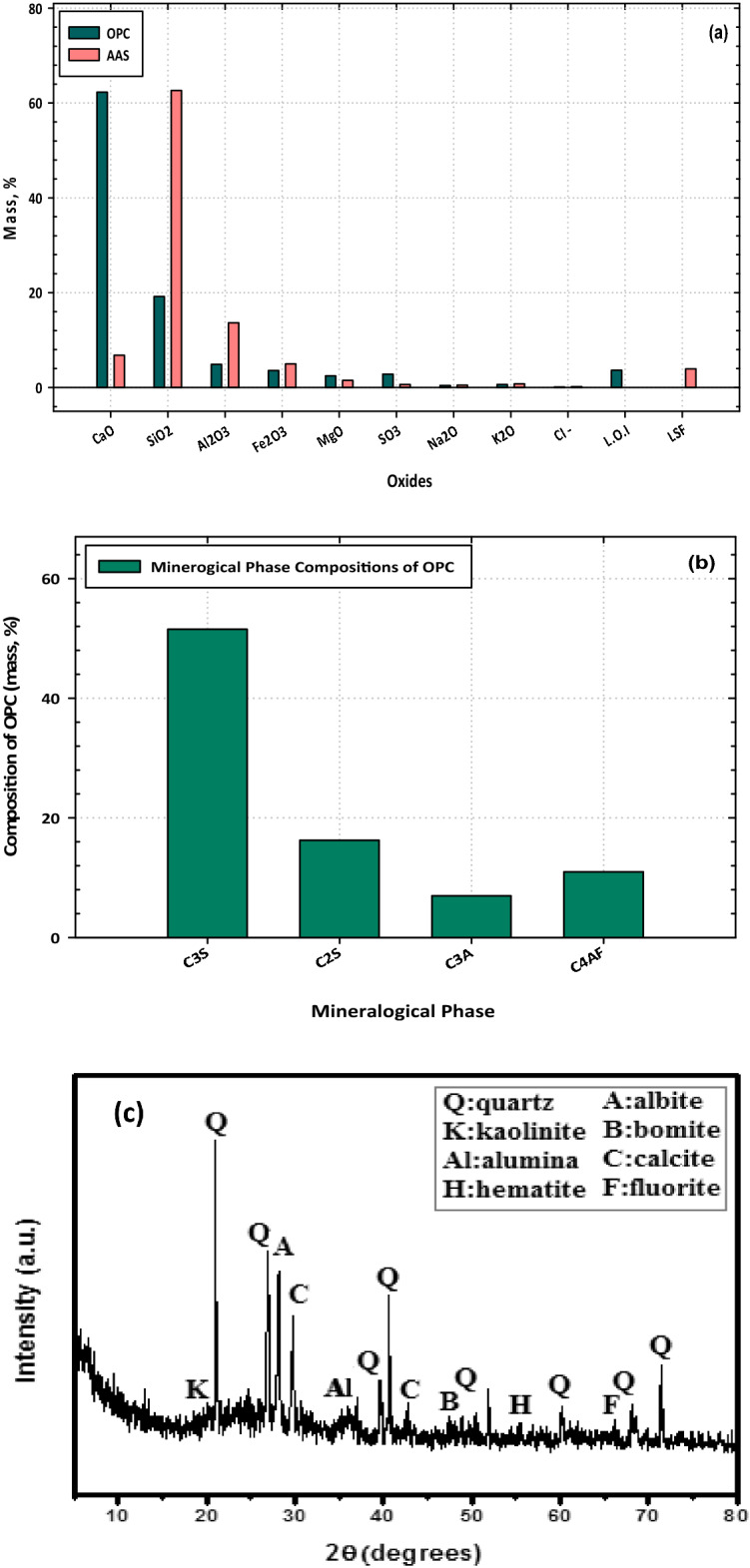
Oxide and mineralogical phase compositions of OPC, (**a**) oxide composition of sludge, and (**b**) mineralogical phase compositions of OPC (mass %) (**c**) XRD patterns activated alum sludge.

The XRF for the chemical oxide compositions of OPC, the mineralogical phase compositions of OPC, and the oxide composition of AAS are outlined in Fig. 2. The pozzolanic activity of activated alum sludge waste (also known as AAS) was evaluated following the TS 25 standards by ensuring that it met the chemical and physical requirements of standards that apply to typical pozzolanic material. This was done to determine whether the waste possessed pozzolanic activity. The TS 25 standard suggests that SiO_2_ + Al_2_O_3_ + Fe_2_O_3_ should be at least 70%, that SO_3_ should be less than 3.0%, that Cl^−1^ should be less than 0.1%, and that the XRF study of AAS highlights the capacity of employing activated alum sludge waste (AAS) as supplemental cementitious materials (SCM) with qualities equal to those of a typical active pozzolanic material. In addition, the standard suggests the fact that XRF analysis was performed on the AAS brings this point home very clearly.

CuFe_2_O_4_ spinel nanoparticles (CFs NPs) with a nanoparticle size of 200 nm were measured by using SEM and created following the previously described approach as follows: utilizing commercial types of reagents and beginning components to lower the economic cost.

The material was ball milled for 40 h to achieve this result, and then it was subjected to 2 h of progressive heating in a muffle furnace at temperatures ranging from 300 to 500 °C. The CFs NPs that were obtained had a size of 50 nm and a value of 39.1 emu/g for their saturated magnetic flux density (Bs), and 4.002 emu/g for their remnant magnetic flux density (Br), and 85.56 Oe for their high coercivity. Additionally, the CFs NPs had a value of 39.1 emu/g for their remnant magnetic flux density (Br) (Hc). The investigation revealed that it preserves a purity level of at least 99.0% throughout its operation. The characteristics and the general properties of CuFe_2_O_4_ spinel nanoparticles are studied since their crystallite size is about 49 nm, the remnant magnetic flux density is about 4.002 B_r_ (emu/g), as well as the saturated magnetic flux density, is about 39.11 B_s_ (emu/g), and finally, its high coercivity is about 85.56 H_c_ (Oe). (CFs NPs). The design and the percentage composition of the different mixtures are outlined in Table 1.

**Table 1:** The percentage composition of the different mixtures and their designations (a) OPC (b) OPC + 5% AAS (c) OPC + 10% AAS (d) OPC + 15% AAS.

Mixtures	OPC	Sludge	Nano (CFs NPs)	Superplasticizer	Water/Cement W/C ratio
A	100.0	0.0	0.0	0.3	0.27
A1	100.0	0.0	0.5	0.3	0.27
A2	100.0	0.0	1.0	0.3	0.27
A3	100.0	0.0	2.0	0.3	0.27
B0	95.0	5.0	0.0	0.3	0.27
B1	95.0	5.0	0.5	0.3	0.27
B2	95.0	5.0	1.0	0.3	0.27
B3	95.0	5.0	2.0	0.3	0.27
C0	90.0	10.0	0.0	0.3	0.27
C1	90.0	10.0	0.5	0.3	0.27
C2	90.0	10.0	1.0	0.3	0.27
C3	90.0	10.0	2.0	0.3	0.27
D0	85.0	15.0	0.0	0.3	0.27
D1	85.0	15.0	0.5	0.3	0.27
D2	85.0	15.0	1.0	0.3	0.27
D3	85.0	15.0	2.0	0.3	0.27

### Water absorption

**Figure 3:**
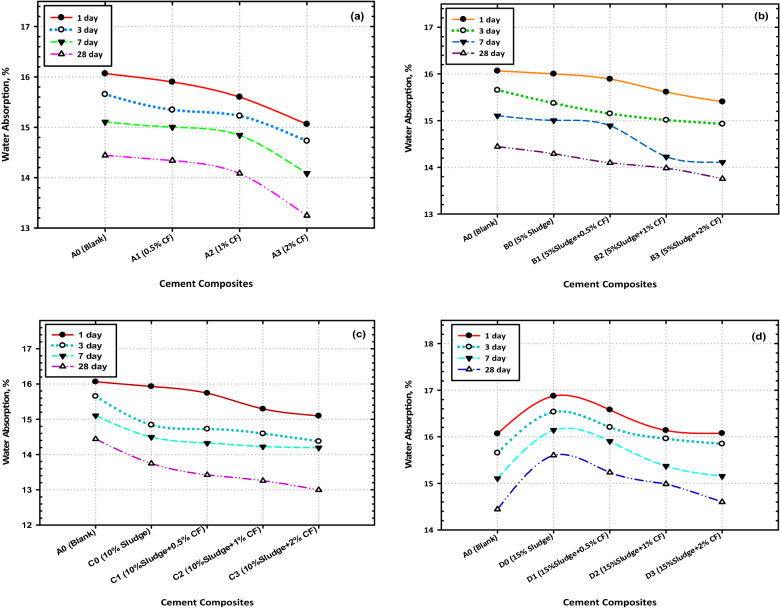
Effect of hydration time on the percentages of water absorption at different cement composites (**a**) OPC (**b**) OPC + 5% AAS (**c**) OPC + 10% AAS (**d**) OPC + 15% AAS.

The results of this experiment are depicted in Fig. 3a, b, c, and d, and they may be summarized in the following way the following conclusions about the water absorption capacities of Mixes A–D were obtained from this experiment. The water absorption (WA) values of all the tested composites decreased as the aging process continued. Blended composites made from Mixes B0 and C0 displayed lower WA percentages as compared to neat OPC (Mix A), while Mix D0 showed comparable or slightly higher values after 28 days. The incorporation of CFs NPs within the OPC–AAS pastes induced the reduction in TP %, which caused declines in the water absorption value of the pastes and the results of the CS, BD, TP, and WA tests indicate that the nanocomposite with the composition 90 OPC–10AAS–2CFs has the optimal composition for application and these test results were found. This is because it demonstrates the best possible physical characteristics in comparison to all the other mixes that were tested throughout most of the testing periods (86.94 Mpa for the CS test; 2.33 g/cm^3^ for the BD test; 35.12% for the TP test; and 12.99% for the WA test after 28 days of hydration)^45^.

### Bulk density

**Figure 4:**
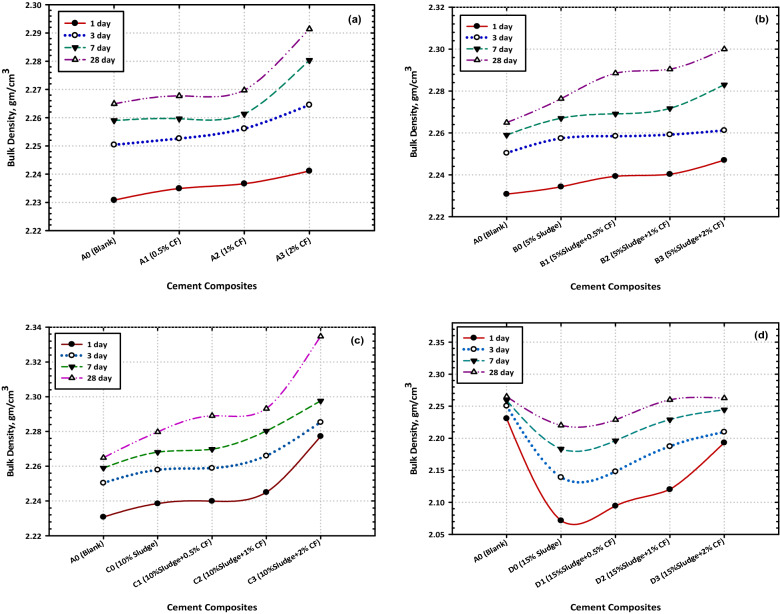
Effect of hydration time on the values of bulk density at different cement composites (**a**) OPC (**b**) OPC + 5% AAS (**c**) OPC + 10% AAS (**d**) OPC + 15% AAS.

Figure 4A, b, c, and d, are a graphical representation of the findings that were obtained by measuring the bulk density (g/cm^3^) of a variety of various mixtures. The values of the bulk density (BD) during the hydration process indicated a steady increase from day 1 to day 28 for each composite that was put through the test. These findings might be explained by the gradual stuffing of pores with accumulated hydration products over time, which allowed for the formation of a dense and thick framework. This occurred as a direct consequence of the buildup of hydration-related compounds. As can be seen in Fig. 4a, the BD values of Mixes B0 and C0 are either more than or comparable to those of the control (Mix A), however, the BD values of Mix D0 are either less than or comparable to the control (Mix A) and the results of this investigation agree with those found in the previous study (CS).

The BD values of the composites that were made from Mixes B0 and C0 increased because of the excessive amount of hydration products that were formed because of the pozzolanic reaction between calcium hydroxide that was liberated from cement clinker hydration and AAS waste. This is a consequence of the pozzolanic reaction that took transpired, which may be explained because these products are piled in most of the open spaces (pores) along with the hardened cement matrix, and the overall porosity of the hardened composites is reduced, which is a topic that will be covered in the subsequent section. At the same time, the BD values of the hardened composites are increased. In the case of the composite built from Mix D0, the number of extra hydration products generated because of the pozzolanic interaction between the available CH and AAS is lower than it is in the case of the composite built from Mixes B0 and C0 for the parameters that were previously indicated and listed in the section on compressive strength.

Because of this, the DB values of this mix were lower than those of (Mixes B0 and C0), but they were still comparable to those of the blank (Mix A) and the most significant discovery is that the hardened nanocomposites, which contain CFs NPs, have higher bulk density values than the materials that served as controls (Mixes A, B0, C0, and D0), and that the densification effect, which is characterized by high bulk density values, increases as the concentration of CFs NPs does as well mentioned in Fig. 4b, c, and d. This finding can be attributed, without a shadow of a doubt, to the activation effect of CFs NPs. CFs act as foreign nucleation centers, which speeds up the hydration process and promotes the formation of an extra amount of C–S–H gel [calcium silicate hydrate (3CaO·2SiO_2_·3H_2_O], CASH, CAH [calcium aluminate hydrate 3CaO·AlO_3_·6H_2_O] (CuSH)^49^.

### Total porosity

**Figure 5:**
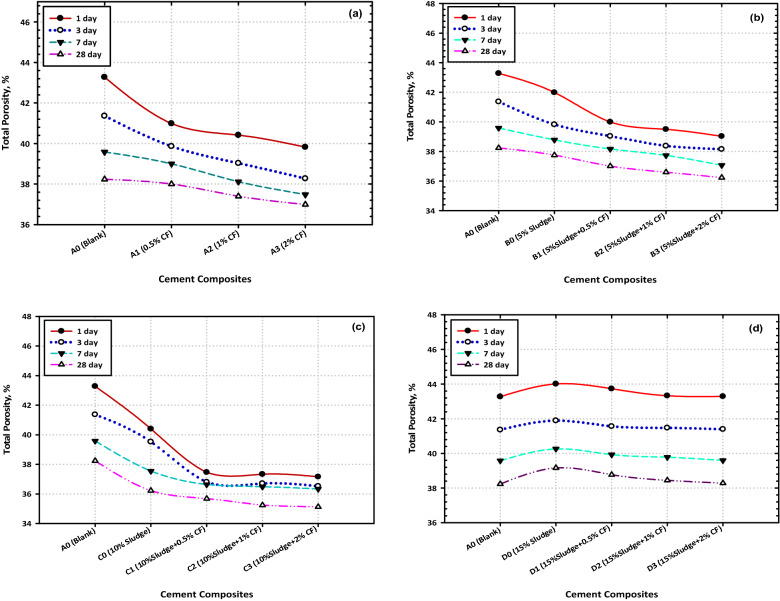
Effect of hydration time on the percentages of total porosity at different cement composites (**a**) OPC (**b**) OPC + 5% AAS (**c**) OPC + 10% AAS (**d**) OPC + 15% AAS.

These extra chemicals congregate in the pores that are accessible, which results in the formation of a structure that is denser, more compact, and with a greater BD. The results of the analysis of the total porosity of the different composites that were tested are presented graphically in Fig. 5a, b, c, and d. As can be seen in Fig. 5a, the values of the TP % decreased steadily during the hydration process (which lasted anywhere from 1 to 28 days) for all the mixes (A–D). This transformation took place as a natural consequence of the hydration process. The stacking of distinct hydrates that grew inside the accessible spaces coupled with the composite matrix is responsible for this decrease in size. Mix B0 and Mix C0 both have TP values that are lower than those of the control sample (Mix A). On the other hand, the TP values for Mix D0 are the same as those of the blank sample. This is an additional point of interest. These findings, along with the findings of the BD and CS, do correlate, and the reasoning behind this correlation may be found in the parts that were just described.

In addition, the incorporation of CFs NPs resulted in a discernible reduction in the total porosity of all the created nanocomposites in comparison to their control, and Fig. 5b, c, and d demonstrate that this reduction in the total porosity percentage is proportional to the quantity of CFs that were mixed in. The integration of CFs NPs results in a decreased percentage of overall porosity. This may be because the CFs NPs perform the functions of both a filler and an activator^50^.

### Thermal resistivity

**Figure 6:**
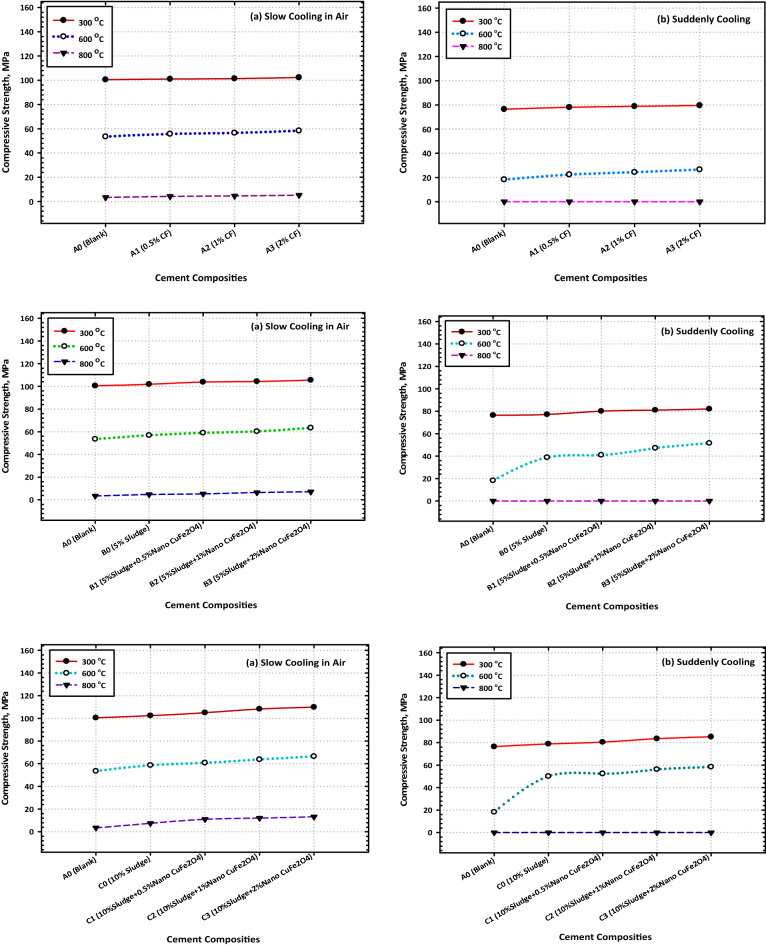

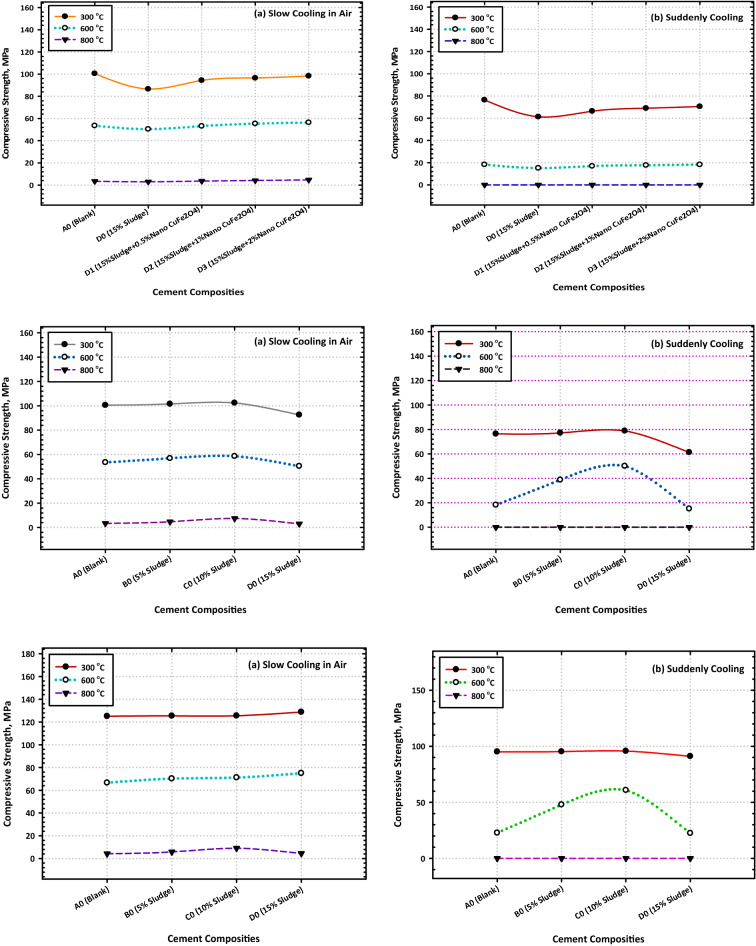
Variation consequence of fired cement composites on the values of compressive strength after (**a**) slow and (**b**) suddenly cooling.

It was investigated what would happen if hardened composites made from OPC, OPC-AAS, and OPC-AAS-CFs were heated to higher temperatures (300, 600, and 800 °C) for 28 days. Figure 6a, and b is a graphical representation of the CS values that were obtained for the various composites after being exposed to fire at various temperatures for 3 h and then being left for gradual cooling in air. These values were obtained after the composites were left for gradual cooling in the air after being removed from the fire. The following is an account of the most important findings obtained. When compared to their recorded values after 28 days of hydration, the compressive strength values of all composites increased significantly during heating up to 300 °C; however, these values decreased significantly during heating up to 600 and 800 °C. The compressive strength values of all OPC–AAS composites increased significantly when compared to those of neat OPC cement (Mix A) at all testing temperatures.

The compressive strength values of all composites increased significantly when compared to those of the following is an example of one explanation that might be correct for these results. The high upgrading of the CS values after being subjected to 300 °C could be attributed to the hydrothermal reaction (internal autoclaving) that takes place between the H_2_O vapors molecules generated from the evaporation of physically adsorbed water inside different pores along with tetrahydrofuran molecules. This reaction takes place between this interaction takes place between the molecules of H_2_O vapors that are produced as a result of the evaporation of water that has been physically adsorbed inside of various pores as well as the hardened cement.

The perfect dispersion of CFs NPs within the composite matrix can be attributed to the obvious improvement in the thermal resistivity of a variety of nanocomposites (especially Mix D3) at 300 °C. This improvement can also be attributed to the composite matrix's efficiency in inducing the formation of large quantities of a variety of hydration products via its nucleation effect and activation of the internal autoclaving. Mix D3 exhibited the greatest degree of improvement in the thermal resistivity mixture. The nanocomposite known as D3 is the one that has demonstrated the largest increase in its thermal resistivity. These products get lodged in the easily accessible gaps (both macropores and micropores) along with the hardened composite, which supports the creation of the hardened matrix, which possesses a high level of resistance to the damaging effects of fire. The decrease in CS values that were observed for all composites after being exposed to 600 °C can be primarily attributed to the thermal degradation of nearly all fundamental products such as CSH composites after firing at 800 °C can be attributed to the complete thermal degradation for all binding centers, in addition to the induction of several cracks along with the composite matrix. This was observed after the composites had been subjected to the temperature.

After exposing the composites to the temperature, this was the resultant observation was made. Figure 6a and b illustrate the change in CS values that occurred in several composites after being heated for 3 h at temperatures of 300, 600, and 800 °C and then having their temperature drop quickly (by immersion in cold water). These composites have CS values that are, beyond a reasonable doubt, significantly lower than those of their equivalents, which were fired at the same temperatures and cooled in the air (slowly). As the firing temperature was increased from 300 to 800 °C, it was observed that the CS of every composite material gradually decreased (Fig. 6a, and b). This was the situation with every one of the composites.

The significant reduction in CS can be attributed to the formation of several cracks as well as the enlargement of the already generated crack (micro-cracks induced during the firing), both of which occurred because of the thermal shock that occurred during the rapid cooling process. Both events took place because of the rapid cooling that took place. In addition, the fact that the micro-cracks were caused by the firing process can be credited for the substantial drop in CS that was seen because of this action. When the temperature was raised from 300 to 600 °C, there was a discernible decline in the compressive strength of every composite that was evaluated, and this decline persisted until it reached 0 °C when the temperature was raised to 800 °C. Despite this, the degree to which loss of strength occurs in blended samples, whether they include CFs NPs, is larger than that which happens in neat Portland cement pastes, whether they contain CFs NPs.

**Figure 7:**
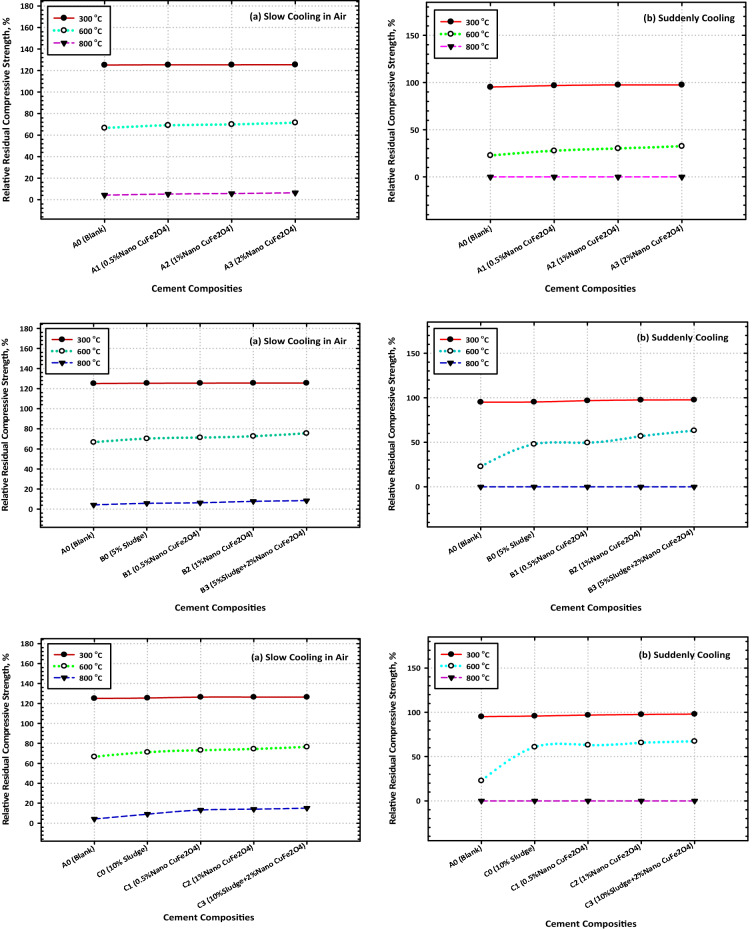

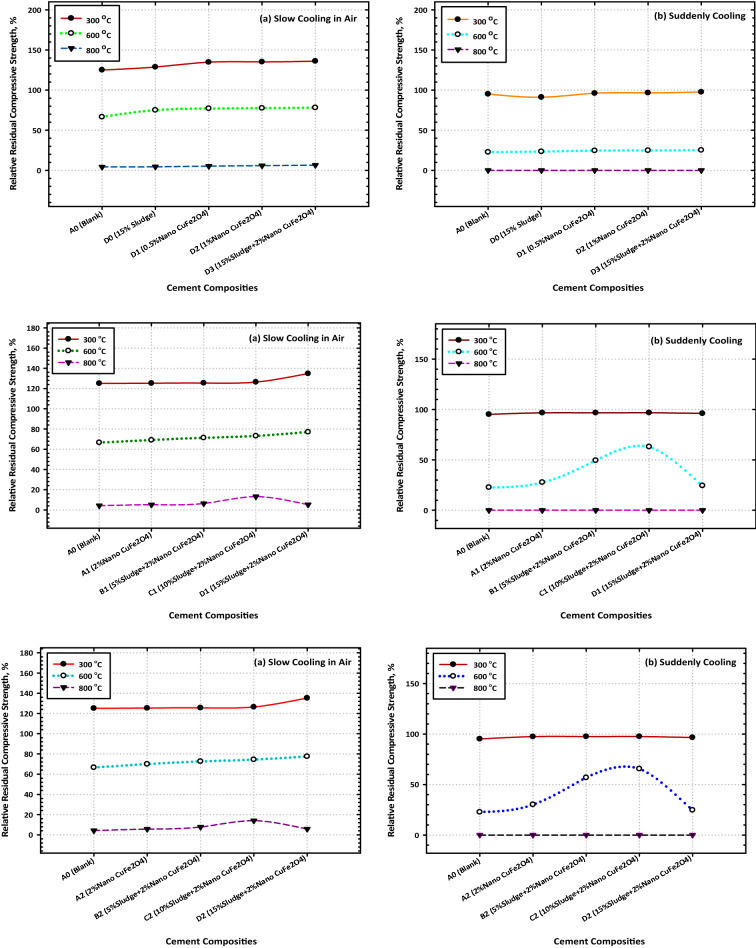

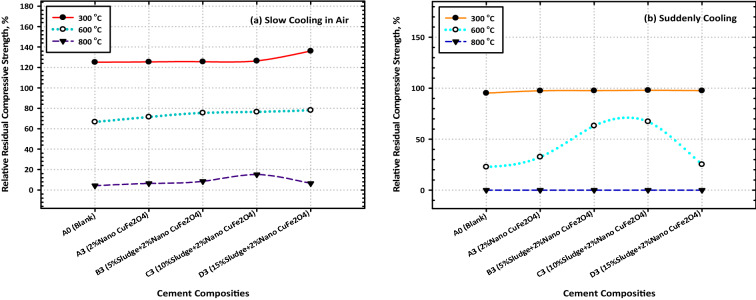
Variation consequence of fired cement composites on the values of the residual compressive strength after (**a**) slow and (**b**) suddenly cooling.

This is the case regardless of whether the blended samples contain CFs NPs. After a certain amount of time has passed, the percent relative compressive strength (RCS) (relative to their CS after 28 days) is displayed in Fig. 7a and b for all burned specimens. The RCS % values that were computed following firing at 300 °C and 900 °C are as follows: 125.16, 125.4, 125.53, and 128.79 for Mixes A–D0, respectively; 125.26, 125.31, and 125.43 for Mixes (A1–A3), respectively; 125.46, 125.58, and 125.61 for Mixes B1–B3), respectively; (Fig. 7a, and b). These data make it abundantly evident that the nanocomposite material that is made up of 85% OPC, 15% AAS waste, and 2% CFs is the one that ought to be selected for use in thermal applications. The fact that this nanocomposite has the highest residual strength (the highest percent RCS) is the best conclusion from the point of view of both the economy and the environment. Figure 7a and b illustrate the percent relative compressive strengths (relative to their CS after 28d) of several different composites after they were subjected to fire and then rapidly cooled.

**Figure 8:**
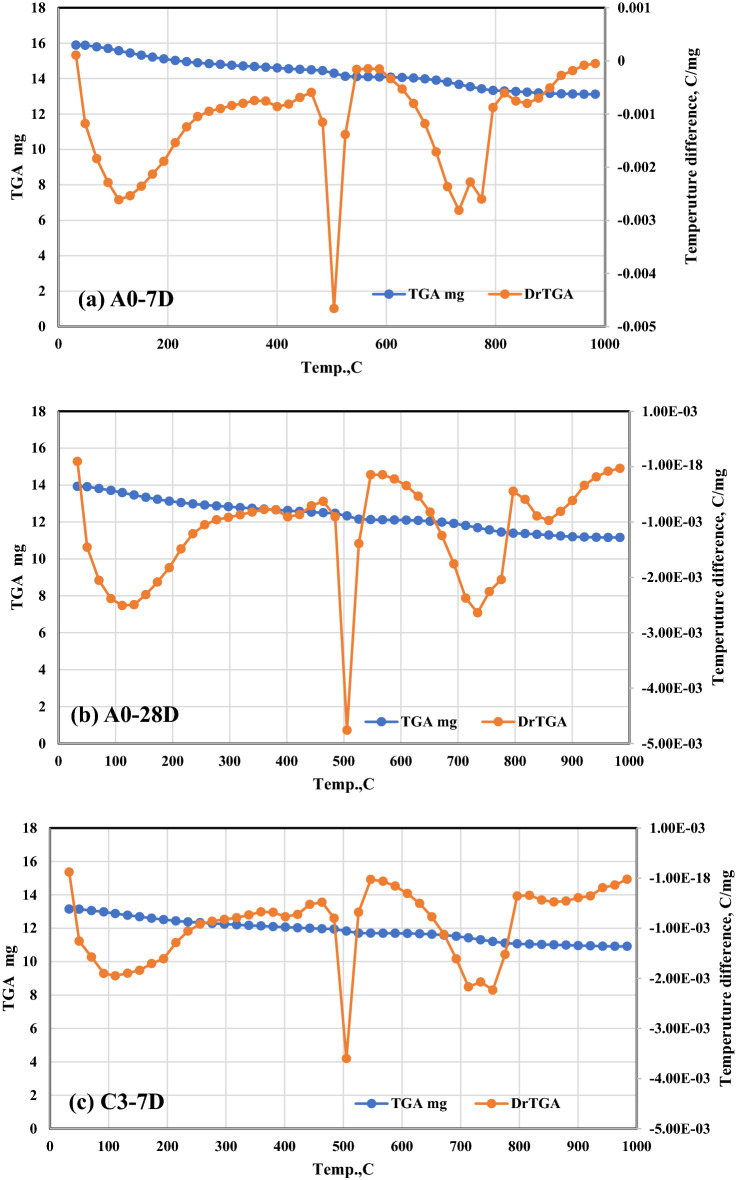

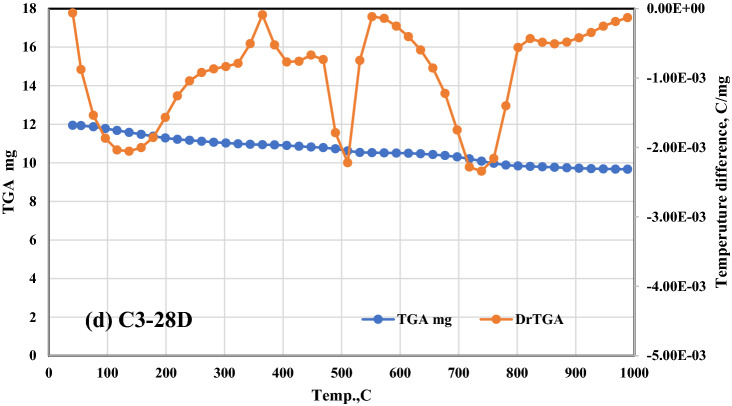
Representative the comparative relationship between TGA and DrTGA (**a**) A0-7D, (**b**) A0-28D, (**c**) C3-7D, and (**d**) C3-28D.

The RCS percent values for these composites are as follows: 95.17, 95.29, 95.79, and 91.12 for Mixes A–D0, respectively, 96.75, 97.45, and 97.62 for Mixes A1–A3, respectively, 96.77, 97.51, and 97.63 for Mixes B1–B3); respectively. After being fired at 300 °C then rapidly cooled. As a result of these observations, the idea is that CFs have a good effect on strengthening the fire resistance of a range of OPC–AAS blended pastes, such as CSHI and CSHII, CAH, CASHs, AFM, AFT, CFSH, and CH, receive additional support. Finally, a noticeable drop in CS levels was observed across the board for all the samples that were analyzed^51^. The comparative study between TGA and DrTGA is represented in Fig. 8a, b, c, and d, and it is noticed that the effect of temperature from 300 to 800 °C through 7 and 28 days is give clear variation between TGA and DrTGA, and the highest effect is noticed in Fig. 8d.

TG–DTA analysis is being used to investigate the thermal resistivity of the FBS. TG–DTA curve for the FBS sample. The initial endothermic peak shown on the DTA curve has its minimum at 85 °C; this is related to a weight loss of about 1% and is caused by the release of hygroscopic water from the FBS. The broad exothermic peak at 315 °C is due to the oxidation of organic matter, and the corresponding weight loss at 425 °C is around 3.27%. We find that the amount of weight loss reflected by the TG curve agrees with the value of volatile matter stated above. It is possible to see a second endothermic peak at a temperature of around 685 °C, the principal cause of which is the oxidation of inorganic compounds and the transition between crystalline phases. Total weight loss at 990 °C is 9.12%, which is quite close to the loss on ignition figure.

### Compressive strength

To undertake an examination of the treated specimens' ensuing mechanical qualities, the compressive strength (CS) values of the specimens were measured at various points throughout the hydration process. This was done so that the values could be compared with one another. In Table 2, the CS values of the hardened composites that were produced by replacing OPC with 0%, 5%, 10%, and 15% of activated alum sludge waste (AAS) (respectively, Mixes A, B0, C0, and D0) are shown. These values were obtained by creating the hardened composites with Mixes A, B0, C0, and D0. These values were achieved by exchanging OPC for waste products generated during the manufacturing of activated alum. In general, the CS values exhibited a consistent pattern with a growing hydration period across the board for every one of the mixes that were put through the testing process.

**Table 2:** Effect of hydration time on the values of compressive strength at different cement composites (a) OPC (b) OPC + 5% AAS (c) OPC + 10% AAS (d) OPC + 15% AAS.

Cement composites	1 day	3 days	7 days	28 days
A0 (Blank)	32.15	52.19	68.35	80.30
A1 (0.5% CF)	33.58	53.64	70.77	80.63
A2 (1% CF)	34.11	54.20	71.32	80.86
A3 (2% CF)	36.88	55.89	72.42	81.56
B0 (5% Sludge)	34.74	53.32	69.67	80.95
B1 (5%Sludge + 0.5% CF)	38.13	55.58	70.30	82.73
B2 (5%Sludge + 1% CF)	39.26	56.06	71.09	83.05
B3 (5%Sludge + 2% CF)	40.39	57.68	72.06	83.99
C0 (10% Sludge)	37.35	56.13	71.00	82.33
C1 (10%Sludge + 0.5% CF)	38.29	58.65	71.90	83.08
C2 (10%Sludge + 1% CF)	44.92	61.07	72.71	85.63
C3 (10%Sludge + 2% CF)	47.16	61.40	73.20	86.94
D0 (15% Sludge)	30.83	49.60	63.34	67.21
D1 (15%Sludge + 0.5% CF)	31.29	50.25	65.11	69.99
D2 (15%Sludge + 1% CF)	32.00	51.83	67.86	71.36
D3 (15%Sludge + 2% CF)	32.10	52.00	68.54	72.23

This continuous increase in the strength values can be generally attributed to the hydration of different phases that are present in Portland cement clinker and the formation of hydration products, primarily in the form of calcium silicate hydrates, calcium aluminate hydrates, alumino ferrite monosulfate hydrate, calcium aluminosilicate hydrate, and calcium ferrite trisulfate, also known as ettringite C6AŠ3H32. In addition, when contrasted with the control (pure OPC) paste, the compressive strength values recorded by the pastes generated by substituting OPC with 5 and 10% of AAS (mass % (Mixes B0 and C0, respectively) recorded the highest values possible at each testing hydration time. These values were determined by comparing the values recorded by the control paste to the values recorded by the pastes generated by substituting OPC with 5 and 10% of AAS (Table 2).

The higher strength values that were reported for these composites are linked to the presence of excessive amounts of nearly amorphous and illcrystalline CSH as the main product. This was obtained through the pozzolanic interaction between AAS waste (alumina and silica phases) and calcium hydroxide, which was obtained from the hydration of cement clinker^52^. These greater strength values are associated with the presence of an excessive quantity of almost amorphous and ill-crystalline CSH as the principal product. This CSH is the culprit behind the higher strength values. These hydrates not only operate as binding sites between the un-hydrated grains that are still present in the system, but they also fill the pores that are present along with the matrix. The matrix is not completely hydrated. On the other hand, increasing the quantity of AAS waste to 15% (Mix D0) causes the CS values to become comparable to, or even lower than, those of blank after 28 days. This occurs because the CS values are affected by the amount of AAS waste. This is since the waste from the AAS gets diluted (Mix A) and this decrease in the CS can be attributed to the dilution effect of PC as a result of its replacement with high percentages of AAS waste, which in turn reduces the amount of Ca(OH)_2_ liberated as a secondary hydration product from C_3_S and -C_2_S phases that required activating the AAS waste. In other words, the amount of Ca(OH)_2_ liberated as a secondary hydration product from C_3_S and –C_2_S phases^53^.

The substitution of PC with significant percentages of AAS waste is to blame for the diluting impact since PC this discovery is compatible with the findings that have previously been published by many studies, and the results of the CS revealed that the optimal replacement ratio of OPC by AAS is 10%; this finding was also verified by the results of the CS. Table 2 provides clarification of the influence that additions of 0.5, 1.0, and 2.0% CFs NPs have on the CS values of neat OPC pastes. The CFs NPs were added to the pastes. There is an illustration of the mixtures A1–A3, B1–B3, C1–C3, and D1–D3. When compared to the values of their references (Mixes A–D0), the addition of different dosages of CFs NPs to OPC or OPC substituted by different masses of AAS waste leads to a significant increase in the compressive strength values of the composite material (Table 2).

This can be seen by comparing the values of the composite material to the values of their references. These findings can be attributed to the nanoparticle characteristics of CF spinel, which include a large specific surface area of 66 m^2^/g, a nano dimension of 50 nm, and, finally, the good distribution of it along with the cement matrix. These characteristics enable it to fill the nano- and micropores existing among different hydration products, which results in a dense and compact structure with higher strength values than those of their control samples (Mix A). Additionally, CFs nanoparticles perform the function of active nucleation centers, which has the effect of accelerating the hydration process of cement grains to produce new and increased quantities of a variety of hydrated phases^54^.

Calcium aluminate hydrates (C_3_AH_6_), calcium aluminosilicate hydrate (Ca–(A)–SH), and calcium ferrosilicate hydrate (like ilvaite, CaFe^2+^) are some examples of the hydrated phases that may be found here CFs nanoparticles since it has been shown that the enforcement impact of CFs NPs increases as the quantity of addition of these NPs increases (from 0.5 to 2%), which is represented as an increase in the CS values for all investigated mixtures. This is a general observation that can be made because of the finding that the quantity of addition of these NPs increases. This improvement can be due to the filling influence that the CFs NPs had on the cement matrix, which led to a decrease in the porosity of the material. In other words, the CFs NPs filled the pores in the cement matrix. In addition to this, the high alkalinity of the composite matrix, which had a pH of more than 12, encouraged the partial ionization of CFs NPs into Cu^2+^ and ferric anion.

These ions have an interaction with Ca(OH)_2_ in the presence of amorphous SiO_2_, which is present in the AAS waste. This interaction results in the generation of an excessive amount of new hydrates, such as copper silicate hydrate Cu_2_Si_2_O_7_(OH)_4_·nH_2_O (CuSH), calcium ferrosilicate hydrate (such as ilvaite, CaFe_2_ + Fe^3+^SiO_7_O(OH), (CFSH), which strongly findings will be supported by the XRD analysis that was performed on a variety of different composites that were put through their paces. In conclusion, the research showed that composite material known as Mix C3, which is comprised of 90% PC, 10% AAS, and 2% CFs NPs, could be regarded as the most advantageous option for use in applications relating to general construction^55^.

This was why it demonstrated the highest CS values when compared to all the other nanocomposites that were tested at nearly all ages. This was the case since it was tested on almost all ages. The substitution of 90% of the OPC with 10% of the AAS contributes to the reduction of costs associated with the disposal of waste (landfill tax), the provision of an alternative use for recycled water-treated plant sludge, without making any assumptions about either its cost or its quality, and the protection of the environment through the conservation of energy and the reduction of the number of harmful gases (CO_2_ and NO_x_) and other air pollutants emitted. There is no question that this composite (90 OPC–10AAS–2 CFs) provides a multitude of benefits, from the point of view of both the economy and the environment^56^.

### Morphology and textural characteristics

**Figure 9:**
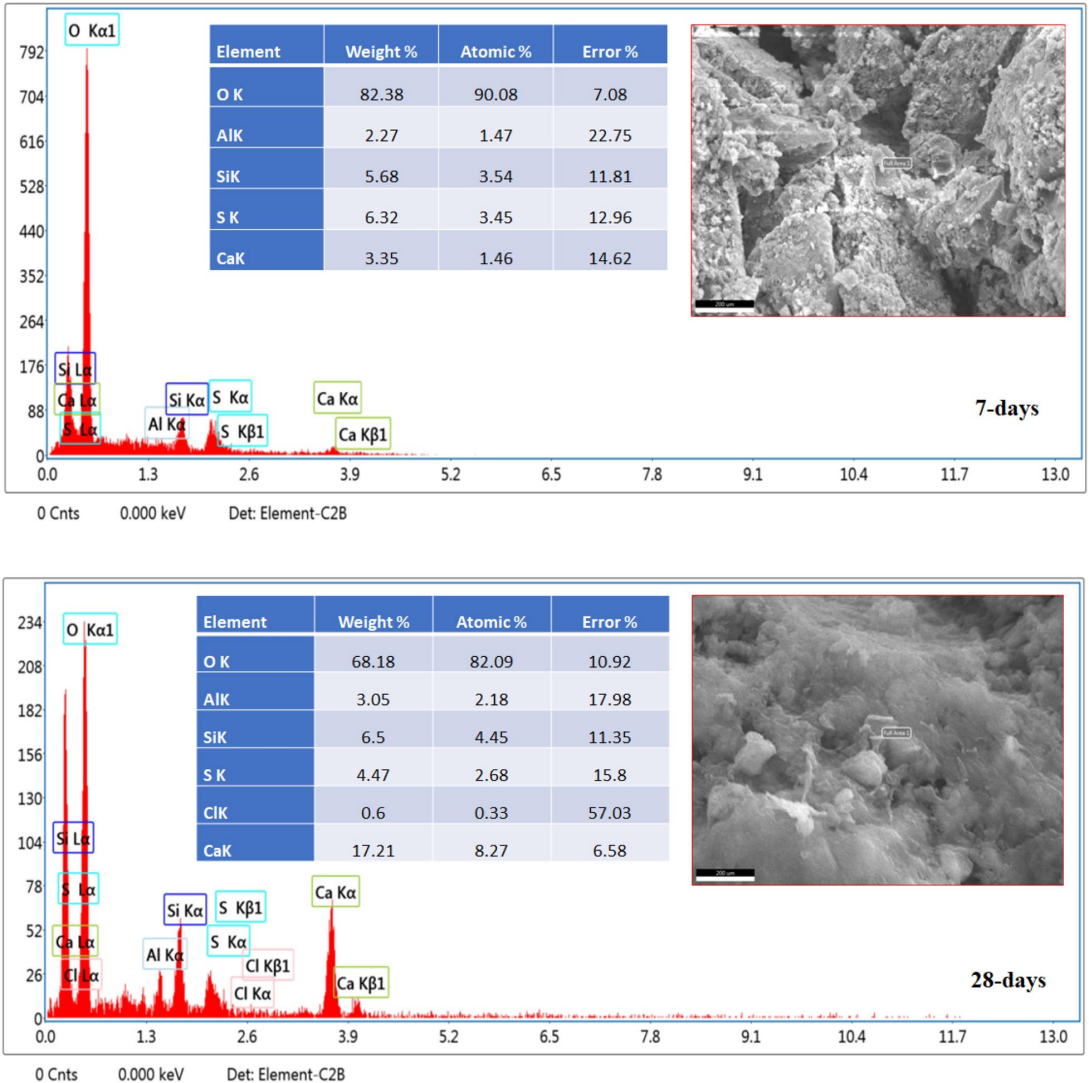
SEM/EDX photographs of OPC at 7-and 28-days.

**Figure 10:**
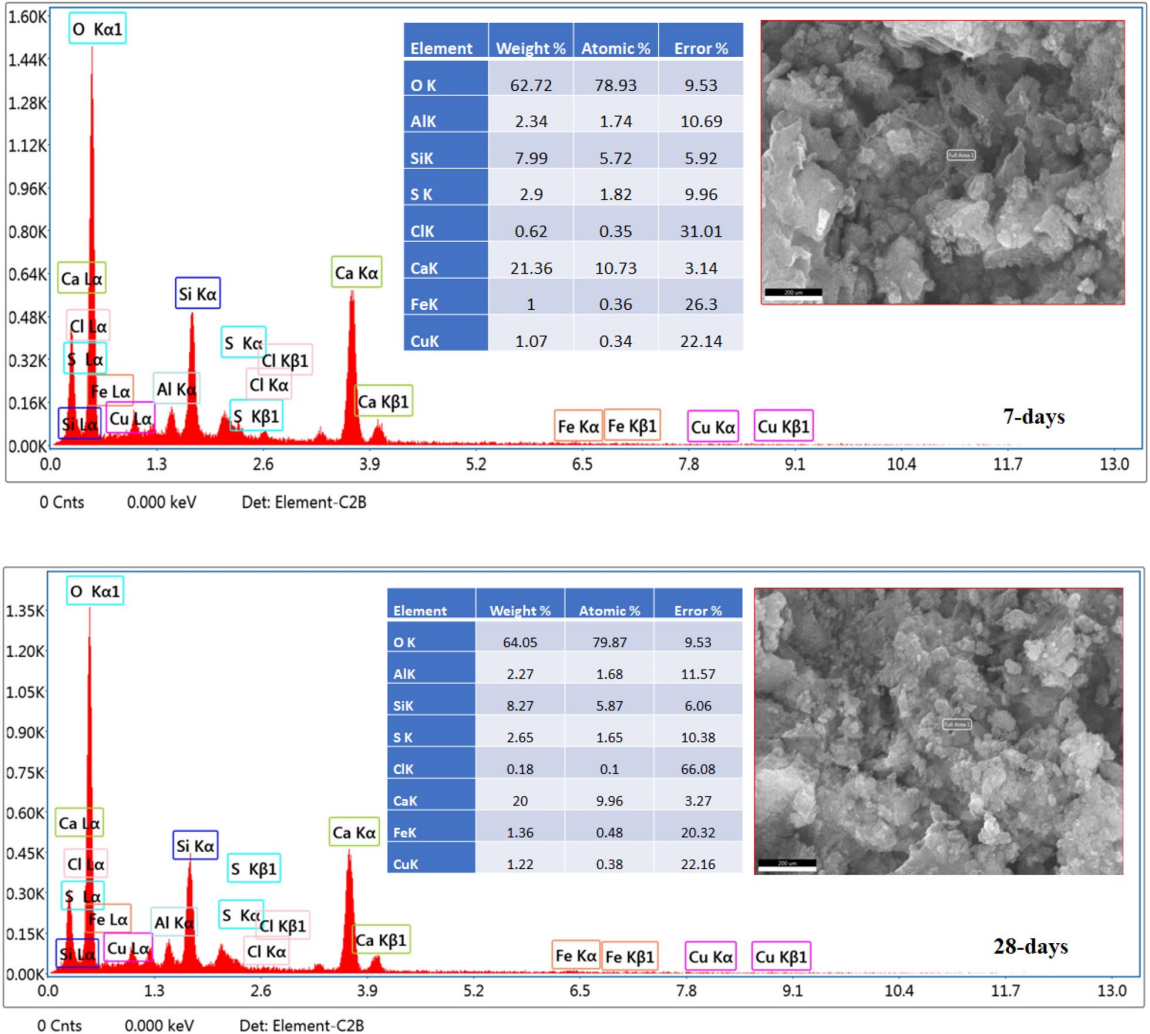
SEM/EDX photographs of OPC-AAS-2 CFs NPs at 7-and 28-days.

The SEM-photographs provided by EDX-analyses of OPC, OPC-AAS-2 CFs NPs at 7 and 28-days of curing are represented in Figs. 9 and 10, respectively. The improvement in hydration products forming escorted by microstructure compaction is a sign of the permanence of hydration with curing intervals at all hardened samples. A low compact microstructure is the mean feature of both OPC -AAS and OPC-AAS-2 CFs microstructures at 7 days, and this is agreeing with the results of compressive strength, which proved that a small number of hydration products, such as C–S–H, as well as a large number of unreacted clinker grains closely, can be detected. After 28-days of hydration, OPC-AAS-2 CFs exhibit microstructure compaction better than those of OPC -AAS at both 7- and 28-days, which is attributed to a dense matrix composed of the excessive generation of force-giving phases (C–S–H, C–A–S–H, and C–F–S–H)^57–60^.

### Corrosion resistance for the blinded material

**Figure 11:**
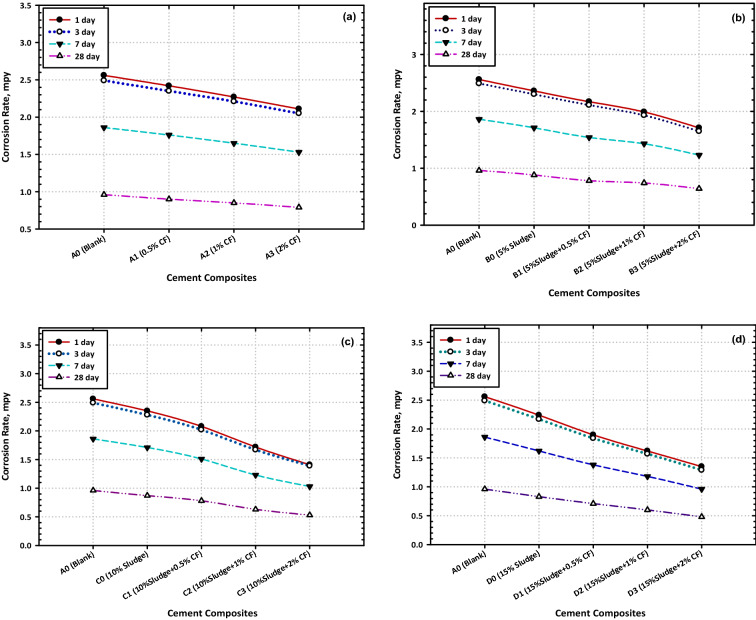
Effect of hydration time on the values of corrosion rates at different cement composites (**a**) Normal cement (**b**) Replace 5% waste (**c**) Replace 10% waste (**d**) Replace 15% water.

The accompanying Fig. 11a, b, c, and d demonstrate how the hydration time it takes for cement to set may have a significant impact on the corrosion rates of mild steel. The investigation revealed that the corrosion rates have decreased over time, which was supported by the obtained data^61–64^. Along with the inherent properties of these combinations, in which the pH values were recorded at the neutral level, which decreased the corrosive influence of the surrounding mild steel medium since the water content of cement will decrease with time, which will result in a reduction in the corrosion rates by diminishing the direct contact between the mild steel surface and the surrounding environment. Both factors will contribute to a reduction in the overall corrosion rates^65–67^. The presence of mild steel in this medium makes it less corrosive than it otherwise would be as a result, the pace at which corrosion occurs slows down; this was proved by the gained results that were provided earlier in the discussion. It was also found that the corrosion rates in the mixtures shown in Fig. 11d are a considerable amount lower than the rates that were reported for the mixtures in Fig. 11a, b, and c correspondingly. This was one of the findings that were shown in Fig. 11d since these data give evidence that increasing the concentration of sludge in the combination has a favorable effect on more than one property, with a reduction in the rates of corrosion being the predominant advantage brought about by this combination^68–73^.

## Conclusion

The physical, mechanical, microstructural, and deteriorating features of a green composite made from OPC and AAS waste were investigated after being subjected to minute amounts of CuFe_2_O_4_ spinel nanoparticles. Making composites from scraps helps save resources and reduce waste. Important findings from this study are summarised in the following paragraphs. All the tests, after which the findings were made public, confirmed that OPC-AAS waste composite could be used as a green construction material. Up to 10% mass replacement of AAS waste for OPC may improve the physical and mechanical properties of hardened composites (CS, TP, BD, and WA%); however, 15% mass substitution of AAS waste has minimal effect on hardened composites. When included in OPC pastes, CuFe_2_O_4_ spinel nanoparticles have the potential to increase the hardness of the paste, which in turn improves the physicomechanical characteristics and thermal resistance of the resulting nanocomposites. The study results support the usage of OPC, AAS waste, and CFs composite materials in common construction settings. Its CS values were the highest of all nanocomposites studied, and as a consequence, its hydration phases also had the highest average values. Most experts agree that the most cost-effective and ecologically friendly choice for construction that will be exposed to high temperatures is a nanocomposite consisting of 85% OPC, 15% AAS waste, and 2% CFs. TGA/DTG and XRD analyses showed that CuFe_2_O_4_ spinel NPs promoted the synthesis of CFSH, AFt, AFM, CASH, and CAHs at elevated concentrations. This was made feasible by the discovery of the NPs with these other chemicals. The NPs' co-location with the predicted locations of these elements provided conclusive evidence of their reality. SEM images demonstrated improved microstructure and mechanical features of the OPC-10 AAS composite after it was heated to 300 °C and CFs NPs were added. To do this, it will be necessary to create a variety of hydration products, including rod crystals, etherate fibers, CASH plates, hexagonal CH sheets, and CuSH gels. Thermal deterioration occurred when the OPC was burned at 800 °C, however it was less severe for most hydration products and micro/micro-cracks than when the OPC was in its pristine condition. Corrosion testing demonstrated that the blended materials' mild steel showed enhanced resistance to corrosion due to the presence of an excessive quantity of sludge.

## Data Availability

All data generated or analyzed during this study are included in this published article.
